# Microbiology and Antimicrobial Resistance of Surgical Site Infection After Inflammatory Bowel Disease Surgery: Prophylaxis–Spectrum Discordance in a Retrospective Cohort from Saudi Arabia

**DOI:** 10.3390/antibiotics15101032

**Published:** 2026-10-16

**Authors:** Ali H. Farsi, Moaz W. Abulfaraj, Ghazal Y. Dhaher, Roaa M. Gaid, Mimoona K. Ayfan, Mariya I. Alrefaei, Mohammed F. Alhazmi, Murad M. Aljiffry

**Affiliations:** 1 Department of Surgery, Faculty of Medicine, King Abdulaziz University, Jeddah 21589, Saudi Arabia; afarsi@kau.edu.sa (A.H.F.); gabdualmoatydaher@stu.kau.edu.sa (G.Y.D.); rhgaid@stu.kau.edu.sa (R.M.G.); msalehayfan@stu.kau.edu.sa (M.K.A.); j1528382@kfshrc.edu.sa (M.I.A.); j1528435@kfshrc.edu.sa (M.F.A.); maljefri@kau.edu.sa (M.M.A.); 2 Department of Surgery, Dr. Soliman Fakeeh Hospital, Jeddah 21461, Saudi Arabia; 3 King Faisal Specialist Hospital and Research Centre, Jeddah 21499, Saudi Arabia; 4 Department of Surgery, Alsalama Hospital, Jeddah 23611, Saudi Arabia

**Keywords:** surgical wound infection, inflammatory bowel disease, Crohn disease, extended-spectrum beta-lactamases, antimicrobial stewardship, antibiotic prophylaxis, antimicrobial resistance, Saudi Arabia

## Abstract

Background/Objectives: Surgical site infection (SSI) after surgery for inflammatory bowel disease (IBD) has not been described microbiologically in the Arabian Peninsula, where extended-spectrum beta-lactamase (ESBL) prevalence is high and prophylaxis remains largely cephalosporin-based. We described the microbiology of SSI after IBD surgery and the extent to which recovered microorganisms were expected to be covered by the prophylaxis administered. Methods: We retrospectively studied consecutive gastrointestinal operations for IBD at a tertiary center in Jeddah, Saudi Arabia (April 2014 to November 2025). Of 118 eligible operations, 8 performed through a dirty/infected wound were reported separately, leaving a primary cohort of 110 operations in 91 patients. The primary outcome was deep incisional or organ/space SSI by CDC/NHSN criteria; models used cluster-robust standard errors for repeated operations. The microbiological analysis was exploratory. Results: SSI followed 33 of 110 operations (30.0%; 95% CI 22.2–39.1), of which 22 (66.7%) were deep or organ/space. Cultures were positive in 23 of 33 infections; because specimens were obtained at clinician discretion, microbiological estimates apply to this subset only. An acquired resistance phenotype was recorded in 10 of 23 (43.5%): ESBL-producing Enterobacterales in 8 and methicillin-resistant *Staphylococcus aureus* in 2. All operations received prophylaxis within 60 min of incision, but five regimens were in use. Expected non-coverage of at least one isolate, assigned from recorded phenotype and intrinsic resistance, was present in 7 of 11 culture-positive infections after cephalosporin-based prophylaxis (63.6%; 95% CI 35.4–84.8) and 0 of 12 after broader-spectrum regimens, with 5 indeterminate. Low preoperative serum albumin and absence of IBD-directed therapy were associated with deep infection after adjustment; emergent surgery was not, although it was in a sensitivity analysis that included the dirty/infected operations. Conclusions: These findings describe prophylaxis–spectrum discordance and support microbiological surveillance and locally informed review of prophylaxis policy rather than empirical broadening.

## 1. Introduction

Surgical site infection remains among the most common complications of gastrointestinal surgery, prolonging hospitalization, increasing cost, and delaying adjuvant or maintenance therapy. Patients undergoing surgery for inflammatory bowel disease are recognized to be at elevated risk, and this association has been characterized in large administrative and registry-based analyses from North America and Europe [1,2,3].

What those analyses do not describe is the microbiology of these infections outside high-income Western settings. Rates of extended-spectrum beta-lactamase production among Enterobacterales differ substantially by region; a national meta-analysis estimated a pooled prevalence of 26.4% in Saudi Arabia [4], and at our own institution more than 95% of ESBL-producing *Escherichia coli* isolates from adult clinical specimens were resistant to third-generation cephalosporins [5]. Surgical antimicrobial prophylaxis guidelines were derived predominantly from populations in which cephalosporin coverage of enteric Gram-negative microorganisms can be assumed. Where that assumption does not hold, the microorganisms recovered from postoperative infections may lie outside the spectrum of the agent administered.

No study has described the causative microorganisms or resistance phenotypes of SSI following surgery for IBD in the Arabian Peninsula. We therefore examined the incidence, anatomical depth, microbiology, and associated factors of SSI in a consecutive surgical IBD cohort at a tertiary center in Jeddah, Saudi Arabia, and described the extent to which the microorganisms recovered were expected to be covered by the prophylaxis administered. This study was designed to describe incidence and associated factors; the microbiological analysis was developed during the analysis phase and is presented as exploratory.

## 2. Results

### 2.1. Cohort Characteristics

**Figure 1:**
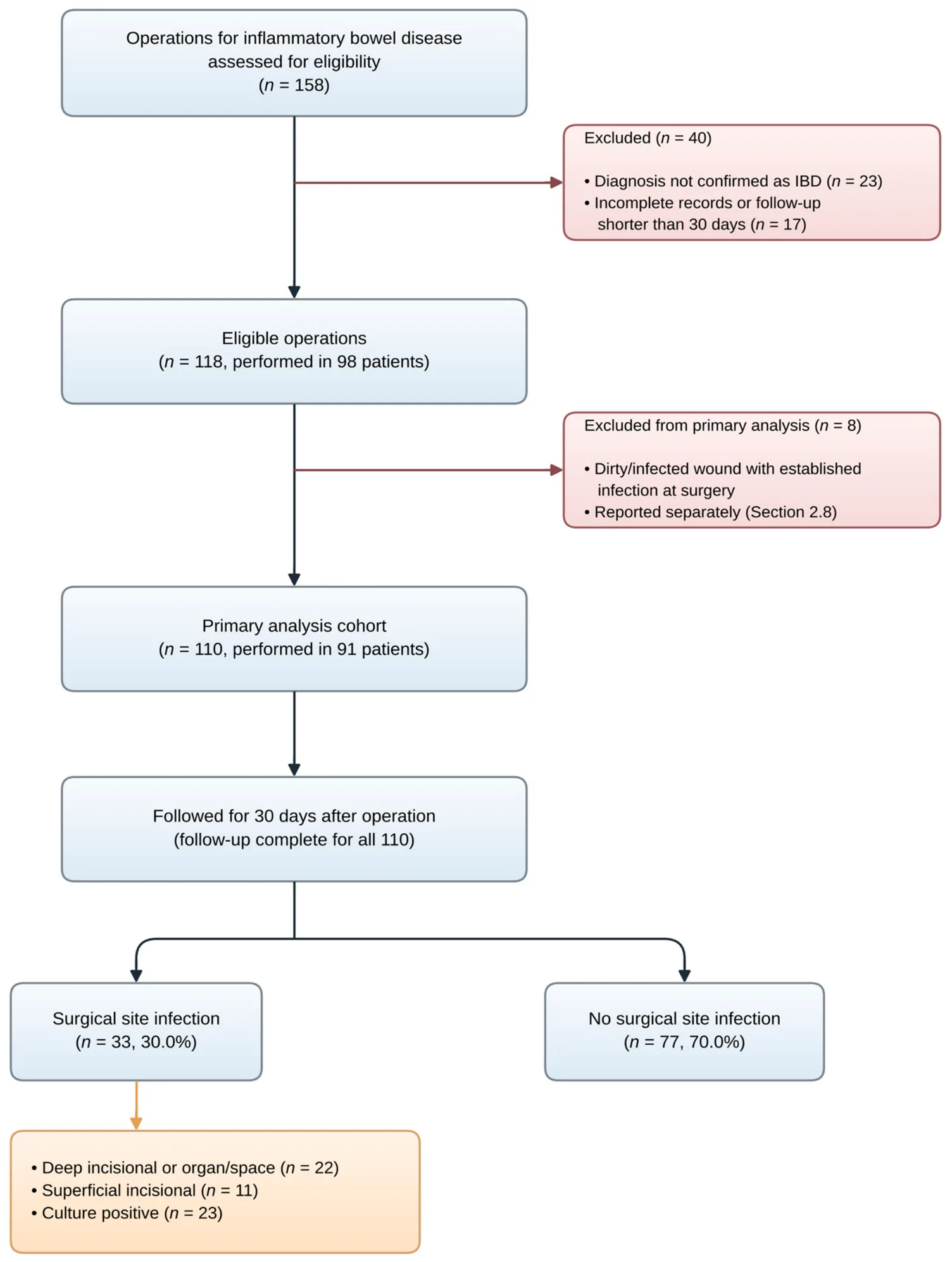
Flow of operations through the study.

Of 158 operations assessed for eligibility, 40 were excluded and 118 were eligible. Eight of the 118 were performed through a dirty/infected wound with established intra-abdominal infection at the time of surgery; because a postoperative infection in these operations cannot be distinguished from persistence of the infection present at operation, they were excluded from the primary analysis and are reported separately in Section 2.8. The primary cohort therefore comprised 110 operations in 91 patients: 74 patients contributed one operation, 15 contributed two, and 2 contributed three (Figure 1). Follow-up to 30 days was complete for every included operation.

The median age at operation was 27 years (IQR 22–41); 66 operations (60.0%) were performed in male patients. Crohn’s disease accounted for 98 operations (89.1%) and ulcerative colitis for 12 (10.9%). Sixty-three operations (57.3%) were performed by an open approach and 47 (42.7%) laparoscopically. Thirty-nine operations (35.5%) were performed in the emergent setting. Thirty-four operations (30.9%) were performed in 2014–2020 and 76 (69.1%) in 2021–2025. Baseline characteristics by SSI status are shown in Table 1.

### 2.2. Incidence and Depth of Infection

SSI was diagnosed after 33 of 110 operations (30.0%; 95% CI 22.2–39.1). Infections were superficial incisional in 11 (33.3% of infections; 10.0% of operations), deep incisional in 17 (51.5%; 15.5%), and organ/space in 5 (15.2%; 4.5%); deep or organ/space infection, the primary outcome, accounted for 22 of 33 infections (66.7%) and 20.0% of all operations (95% CI 13.6–28.4). Median length of stay was 7 days (IQR 5–11) overall, and was longer following operations complicated by SSI (12 days, IQR 7–20) than those without (5 days, IQR 4–8; *p* < 0.001).

### 2.3. Microbiology

**Figure 2:**
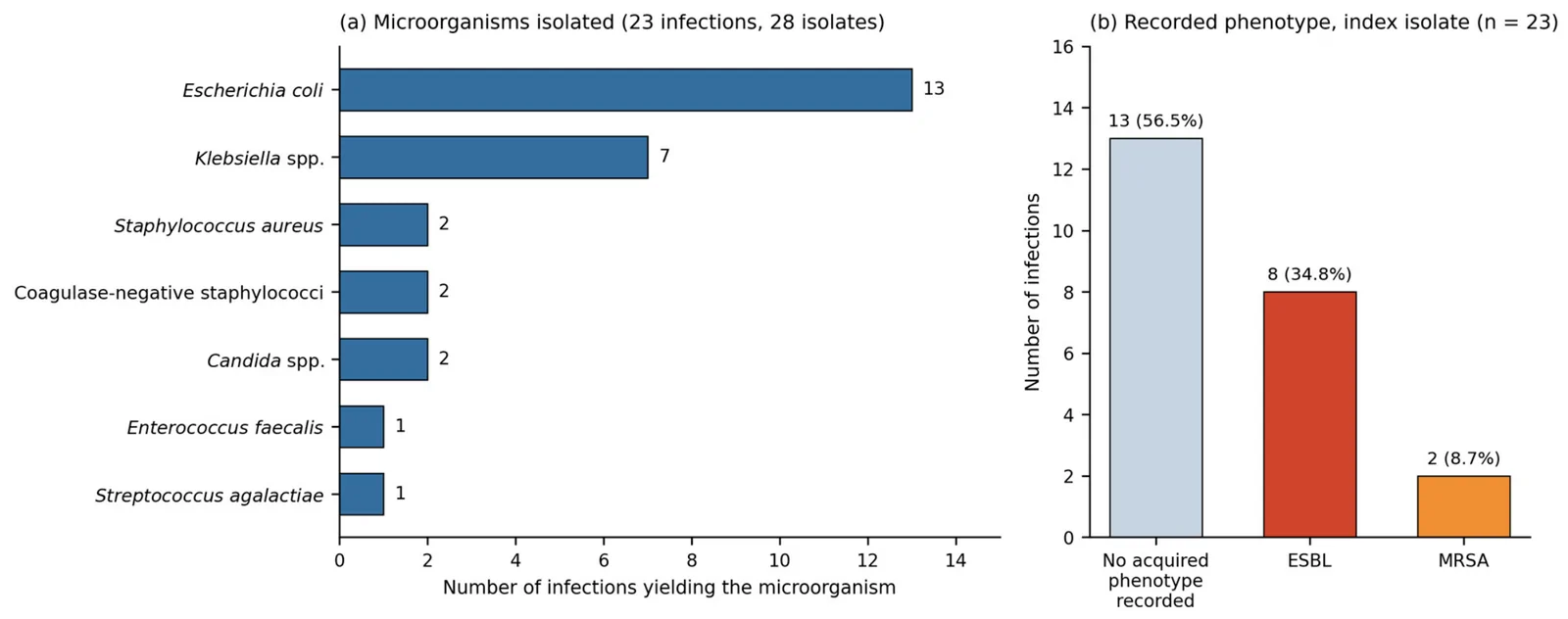
Microorganisms isolated from culture-positive surgical site infections (**a**) and recorded resistance phenotypes (**b**). ESBL, extended-spectrum beta-lactamase; MRSA, methicillin-resistant *Staphylococcus aureus*.

Cultures were positive in 23 of the 33 infections (69.7%; 95% CI 52.7–82.6). Of the remaining 10, a culture was recorded as obtained with no growth in 6 and no culture was recorded in 4. Specimens were obtained at the discretion of the treating clinician; no institutional protocol governed which infections were cultured, and all microbiological estimates below therefore apply to the culture-positive subset only. Five of the 23 culture-positive infections (21.7%) were polymicrobial. Isolates were taken as reported by the laboratory; no formal criteria distinguished causative from colonizing or contaminating microorganisms, and the two infections yielding only a coagulase-negative staphylococcus, both from wound swabs, are flagged as possible contaminants in Section 4.4. Enterobacterales predominated among the 28 isolates: *Escherichia coli* was recovered from 13 infections and *Klebsiella* species from 7, with *Staphylococcus aureus*, coagulase-negative staphylococci, *Candida*, *Enterococcus faecalis*, and *Streptococcus agalactiae* accounting for the remainder (Table 2; Figure 2).

An acquired resistance phenotype was recorded for the index isolate in 10 of 23 culture-positive infections (43.5%; 95% CI 25.6–63.2). ESBL production was recorded in eight (34.8%; 95% CI 18.8–55.1), comprising seven *E. coli* and one *Klebsiella oxytoca*, and methicillin resistance in the two *S. aureus* index isolates; no carbapenemase producer was recovered in the primary cohort. “No resistant phenotype recorded” denotes that the laboratory report identified none of these acquired phenotypes for the index isolate; it does not establish susceptibility to the prophylactic agent administered.

### 2.4. Antimicrobial Prophylaxis and Expected Coverage of Isolates

Every operation received a preoperative antimicrobial dose within 60 min of incision, but the regimen was chosen by the operating surgeon without a written protocol (Section 4.5). Five regimens were administered (Table 3): cefazolin or ceftriaxone with metronidazole in 65 operations (59.1%, cephalosporin-based), and ciprofloxacin with metronidazole, piperacillin–tazobactam, or meropenem in 45 (40.9%, broader-spectrum). All regimens other than piperacillin–tazobactam and meropenem included metronidazole, so anaerobic coverage was present throughout and the coverage assessment below concerns aerobic microorganisms. Regimen selection tracked the clinical circumstances of the operation: the proportion of emergent operations rose from 20% with cephalosporin-based regimens to 76% with piperacillin–tazobactam or meropenem.

Redosing was indicated by institutional practice for operations exceeding four hours, which applied to 54 of 110 operations (49.1%); whether redosing was actually given in each case could not be established retrospectively.

Infection followed 21.5% of operations receiving a cephalosporin-based regimen and 42.2% of those receiving a broader-spectrum regimen. This comparison is presented for description only: the broader-spectrum group contained 26 of the 39 emergent operations (66.7%), the agent was selected on the basis of the surgeon’s assessment of contamination, and the comparison is therefore confounded by indication in a way that adjustment cannot resolve. We draw no inference about the effectiveness of either regimen from it.

Expected coverage of each culture-positive infection by the regimen administered was assessed against every reported isolate, using the recorded acquired-resistance phenotype together with known intrinsic resistance; agent-specific susceptibility results were not uniformly retrievable, and isolates whose coverage could not be determined from these criteria were classified as indeterminate (Section 4.4). The same regimen-specific criterion was applied to every infection in both groups. Among the 11 culture-positive infections following a cephalosporin-based regimen, at least one isolate was expected not to be covered in 7 (63.6%; 95% CI 35.4–84.8): 5 ESBL-producing Enterobacterales, one of which was co-isolated with *Enterococcus faecalis*, and 2 methicillin-resistant *S. aureus*. The remaining four were classified as covered. Among the 12 culture-positive infections following a broader-spectrum regimen, none was expected not to be covered (0%; 95% CI 0–24.2); 7 were classified as covered and 5 as indeterminate, because the susceptibility of an ESBL-producing isolate to ciprofloxacin, or of a coagulase-negative staphylococcus or *Candida* isolate to the agent given, could not be determined from the recorded phenotype. The difference in expected non-coverage between the two groups was *p* = 0.001 with indeterminate infections counted as covered, *p* = 0.013 with indeterminate infections excluded, and *p* = 0.41 with indeterminate infections counted as not covered. The classification of every culture-positive infection is shown in Table 4.

### 2.5. Factors Associated with Infection

On univariable analysis (Table 1), open approach, diabetes mellitus (5 patients), and older age were associated with SSI; emergent surgery was not associated with any SSI (35.9% vs. 26.8%, *p* = 0.39) but was associated with deep or organ/space infection (30.8% vs. 14.1%, *p* = 0.047). Preoperative albumin was lower following operations complicated by infection, and infection followed 37.7% of operations with an albumin of 35 g/L or less compared with 20.4% above that threshold, a difference at the margin of significance (*p* = 0.061). Sex, disease type, smoking, wound classification, stoma formation, perianal disease, ASA class, and operative duration were not associated with infection.

Patients who had received any IBD-directed therapy had a lower rate of SSI than those who had not (23.4% vs. 45.5%; *p* = 0.025), but no individual drug class was associated with infection when examined separately (Table 1), and untreated patients underwent emergent surgery more often than treated patients (45.5% vs. 31.2%).

### 2.6. Multivariable Analysis

All 110 operations contributed to the multivariable models, comprising 33 infections and 22 deep or organ/space infections. Standard errors were clustered by patient to account for the 17 patients who contributed more than one operation; a generalized estimating equation with exchangeable correlation gave estimates that differed by less than 5% and is not shown separately. For deep or organ/space infection, preoperative albumin 35 g/L or less (aOR 3.59, 95% CI 1.17–11.04) and absence of IBD-directed therapy (aOR 3.58, 95% CI 1.29–9.97) were associated with infection after adjustment, whereas emergent surgery (aOR 2.39, 95% CI 0.79–7.22) and open approach were not; with any SSI as the outcome, only absence of IBD-directed therapy reached significance (Table 5). Preoperative albumin reached significance only after adjustment, the univariable comparison having fallen just short; its stability is examined in Section 2.7. The four predictors were fitted on 22 events, 5.5 events per variable, which is below the conventional guide of ten; model diagnostics were nonetheless acceptable (variance inflation factors 1.01–1.05; Hosmer–Lemeshow *p* = 0.50; area under the curve 0.77), and Firth penalized estimation, which corrects for small-sample bias, gave slightly attenuated but similar odds ratios (Table 6). Because absence of IBD-directed therapy is itself associated with emergent presentation, a three-predictor model omitting it was also fitted; preoperative albumin was retained and emergent surgery remained non-significant (Table 6).

### 2.7. Sensitivity Analyses

Five sensitivity analyses were performed for the primary outcome (Table 6). Including the 8 dirty/infected operations, all of which were emergent and all of which were followed by infection (118 operations, 30 events), made emergent surgery the strongest association (aOR 3.92, 95% CI 1.36–11.28) while leaving the albumin and IBD-therapy estimates similar; the association between emergent surgery and deep infection in this cohort is therefore driven in large part by operations on an already-infected field. Restricting the primary cohort to the first operation per patient (91 operations, 19 events) preserved the association with absence of IBD-directed therapy (aOR 3.57, 95% CI 1.14–11.16), whereas the association with preoperative albumin weakened and lost significance (aOR 2.92, 95% CI 0.80–10.59). Adding study period (2014–2020 versus 2021–2025) to the model left the direction and significance of the covariate estimates unchanged, and the crude infection rate did not differ between periods (29.4% versus 30.3%; *p* = 1.0); cephalosporin-based regimens were used in 73.5% of operations in the earlier period and 52.6% in the later period, and an acquired resistance phenotype was recorded in 2 of 6 culture-positive infections in the earlier period and 8 of 17 in the later period. Restricting the cohort to patients with Crohn’s disease (98 operations, 28 infections) left the direction of the albumin and IBD-therapy associations unchanged, although neither retained statistical significance.

### 2.8. Dirty/Infected Operations

The eight operations performed through a dirty/infected wound are reported separately (Table 7). All were emergent, all involved established intra-abdominal infection at the time of surgery, and all received a non-cephalosporin regimen (four received piperacillin–tazobactam, three ciprofloxacin with metronidazole, and one meropenem), in which case the agent may have served as treatment of established infection rather than prophylaxis. All eight were followed by a postoperative infection meeting the surveillance definition of SSI, deep incisional in two and organ/space in six. Seven were culture-positive; three of the seven yielded an isolate expected not to be covered by the agent given (*E. faecalis* after ciprofloxacin, *Enterococcus faecium* after meropenem, and a carbapenemase-producing *E. coli* after piperacillin–tazobactam), two were classified as covered, and two as indeterminate. Whether these infections represent new postoperative events or persistence of the infection present at operation cannot be determined from the records, which is why they were excluded from the primary analysis. With these operations included, the overall infection rate for all 118 eligible operations was 41 of 118 (34.7%; 95% CI 26.8–43.7).

## 3. Discussion

In this consecutive surgical IBD cohort, excluding operations with established infection at the time of surgery, 30% of operations were followed by SSI, and two-thirds of those infections were deep incisional or organ/space rather than superficial. More than two-fifths of culture-positive infections involved an isolate with a recorded acquired resistance phenotype. The absence of IBD-directed therapy was associated with deep or organ/space infection across all model specifications, and preoperative hypoalbuminemia in most; emergent surgery was not associated in the primary cohort, and its association in the sensitivity analysis that included dirty/infected operations reflects those operations. The association with preoperative hypoalbuminemia is consistent with previous reports in gastrointestinal and Crohn disease surgery [6,7,8], although it reached significance only after adjustment and weakened in the one-operation-per-patient analysis, and is best regarded as suggestive.

### 3.1. Incidence in Context

The overall infection rate exceeds that reported in large Western registry analyses of colorectal and IBD surgery [1,2,3]. Three features of this cohort explain much of the difference. First is the depth distribution: superficial incisional infection accounted for only 10.0% of operations, and the rate is driven by deep and organ/space infection, which argues against ascertainment of minor wound problems as the explanation. Second, the case mix is Crohn’s-predominant, with a high proportion of penetrating disease, and median operative duration exceeded 3.5 h. Third, more than a third of operations were performed emergently, and the cohort was drawn from a tertiary referral population with a high proportion of complicated disease. Operations with established infection at the time of surgery, which would have raised the rate further, have already been set aside. Comparison with registry benchmarks should therefore be read against this case mix rather than as evidence of a difference in the quality of care.

### 3.2. Prophylaxis–Spectrum Discordance

The central descriptive observation is a discordance between the agents administered and the microorganisms recovered. Prophylaxis timing in this cohort was good: every operation received a dose within 60 min of incision, consistent with guideline recommendations [9,10], although timing alone does not establish adequate exposure (Section 3.5). Yet among culture-positive infections following cephalosporin-based prophylaxis, nearly two-thirds yielded an isolate that the agent was not expected to cover, each on the basis of an acquired mechanism, ESBL production or methicillin resistance, or the intrinsic resistance of *Enterococcus* to cephalosporins. Two qualifications should be noted. The first is that current CLSI and EUCAST guidance reports cephalosporin susceptibility from the measured MIC rather than from ESBL status, so ESBL production does not in itself establish resistance to every cephalosporin; because agent-specific results were not retrievable, classifying ESBL producers as not expected to be covered by cefazolin or ceftriaxone rests on the recorded phenotype rather than on measured MICs. Local data support that classification: more than 95% of ESBL-producing *E. coli* at our institution were ceftriaxone-resistant [5]. The second is that the estimate rests on 11 infections.

This observation is consistent with infections after a given regimen being enriched for microorganisms that the regimen does not cover; establishing a causal selection effect would require a different design. Regimens were not randomly assigned; the microbiological question was developed during analysis; cultures were obtained at the clinician’s discretion and were more likely to have been taken from infections that failed initial management; and the pattern could equally reflect prior antimicrobial exposure, preoperative colonization with resistant microorganisms, or the severity of the underlying disease, none of which could be measured retrospectively. We therefore describe the finding as prophylaxis–spectrum discordance and treat it as hypothesis-generating. The complementary observation, that no culture-positive infection following a broader-spectrum regimen yielded an isolate expected not to be covered, is subject to the same caveats and additionally to a large indeterminate fraction; it was statistically significant only when indeterminate infections were counted as covered or excluded, and is reported for completeness rather than as support for the conclusions.

A second observation concerns the absence of a standardized regimen. Five prophylaxis regimens were in use across the 110 operations of the primary cohort, selected at the discretion of the operating surgeon. No written criteria governed the choice, which in practice tracked the surgeon’s assessment of urgency and contamination, and it shifted over the study period: cephalosporin-based regimens were used in 74% of operations in 2014–2020 and 53% in 2021–2025. Discretionary selection is not in itself inappropriate, but it leaves the institutional regimen unauditable against any benchmark and precludes the before-and-after evaluation that would be needed to test whether a change in policy improved outcomes. A written protocol is a prerequisite for the local antibiogram review we propose, and established guidance on agent selection, dosing, and intraoperative redosing is available to inform it [9,11].

These data do not support empirical broadening of prophylaxis. Routine carbapenem or piperacillin–tazobactam prophylaxis would accelerate the very resistance that generated the finding, and nothing in our own data would justify such a change: infection was twice as frequent after broader-spectrum regimens, but that group contained two-thirds of the emergent operations, and the agent chosen records the surgeon’s assessment of contamination rather than an intervention that can be evaluated observationally. They also do not show that broader prophylaxis reduces resistant infection or improves outcomes. What they do show is that the microorganisms recovered from SSI after IBD surgery in this setting are frequently not those that prophylaxis guidelines assume, and that warrants surveillance rather than escalation: institutional review of prophylaxis policy against the local antibiogram, and routine microbiological surveillance of SSI, so that any change rests on local data. Prospective, protocol-driven culture sampling with agent-specific susceptibility testing and standardized follow-up would be required before any change in prophylaxis policy could be recommended.

### 3.3. The Apparent Protective Effect of IBD Therapy

Patients who had received IBD-directed therapy had a lower rate of SSI, and this association persisted in the multivariable models. This appears to contradict a substantial body of literature demonstrating that corticosteroids and anti-tumor necrosis factor agents increase postoperative infection risk [12,13,14,15,16], although a recent large single-center series found no excess risk attributable to perioperative biologic exposure once disease activity and concomitant therapy were accounted for [17], and pooled analysis of two randomized trial cohorts identified steroid or immunosuppressant use as an independent risk factor for incisional infection after gastroenterological surgery [18]. We interpret it as confounding by indication rather than a treatment effect, for two reasons. First, no individual drug class was associated with infection when examined separately; the signal rests entirely on the composite of having received any therapy. Second, untreated patients underwent emergent surgery more often than treated patients, and untreated status in this population identifies the newly diagnosed, the non-adherent, and those presenting acutely with perforation or abscess. Host factors beyond drug exposure may also contribute; the ileal mucosa-associated microbiota has been shown to predict infection after ileocolonic resection for Crohn disease [19].

This finding carries a methodological lesson. Single-center series that treat ‘received IBD therapy’ as an exposure without accounting for the urgency and indication of surgery will reproduce this artifact. We have retained the variable in the primary model for transparency, but report alongside it a three-predictor specification omitting it; we do not present absence of therapy as an independent clinical risk factor.

### 3.4. Operative Factors

Open approach was associated with infection on univariable analysis but not after adjustment, which is consistent with open surgery being selected for the more contaminated and more urgent cases rather than causing infection in itself. Randomized comparisons in small-bowel Crohn disease have reported fewer wound infections after laparoscopic than open resection, although the difference did not reach significance [20]. Emergent surgery was not associated with deep infection in the primary cohort; it became the strongest association only when the dirty/infected operations were included, so part of what “emergent” captured in the full cohort was operation on an already-infected field.

### 3.5. Strengths and Limitations

The principal limitation of this study is the small number of events underlying the central estimate. The expected non-coverage proportion rests on 11 culture-positive infections following cephalosporin prophylaxis, and its confidence interval spans 35% to 85%. Agent-specific susceptibility results were not uniformly retrievable, so coverage was classified from the recorded acquired-resistance phenotype and known intrinsic resistance rather than from susceptibility testing; “no phenotype recorded” does not establish susceptibility, and five infections could not be classified. Cultures were obtained at the clinician’s discretion rather than by protocol, 10 of 33 infections had no positive culture, and the microbiological estimates apply only to the culture-positive subset, in which resistant microorganisms are likely over-represented. Prior antimicrobial exposure and preoperative colonization were not retrievable and could not be adjusted for. The study spans nearly a decade, over which resistance patterns, surgical practice, IBD therapy, and laboratory methods may have changed; the infection rate was stable across the two halves of the period and covariate estimates were unchanged by adjustment for period, but a shift away from cephalosporin-based regimens over time was evident. Seventeen patients contributed more than one operation; clustering had little effect on the estimates, but the one-operation-per-patient analysis weakened the albumin association. Events per variable were 5.5, below the conventional guide, and although diagnostics and Firth estimation were reassuring, the adjusted estimates should be regarded as provisional pending confirmation in a larger or multicenter cohort. The eight dirty/infected operations all involved established infection at operation and are reported separately. Specimens included wound swabs, whose recovery of colonizing flora is well recognized, and no formal criteria distinguished causative from colonizing microorganisms. Because routine clinic review took place at about two weeks, a superficial infection arising after that visit and managed entirely outside the institution could have escaped ascertainment; deep and organ/space infections, which required readmission or intervention, are unlikely to have been missed. The retrospective single-center design constrains generalizability. Dose, weight-based adjustment, the duration of postoperative antimicrobial administration, and the frequency of intraoperative redosing could not be established, so factors other than spectrum may have contributed to individual failures; median operative duration exceeded the recommended redosing interval for cefazolin [9], so inadequate redosing remains an unmeasured potential contributor. The strengths of this study are the consecutive design, complete 30-day follow-up, the per-isolate reporting of microbiology, and the reporting of a setting for which no comparable data exist.

## 4. Materials and Methods

### 4.1. Study Design and Setting

This was a retrospective cohort study conducted at King Abdulaziz University Hospital, a tertiary academic referral center in Jeddah, Saudi Arabia, serving the western region of the country. The study was reported in accordance with the STROCSS 2021 criteria [21]. It was conducted in accordance with the ethical principles of the Declaration of Helsinki, and the protocol was reviewed and approved by the Institutional Review Board of King Abdulaziz University, Jeddah (reference 239-25). The requirement for informed consent was waived by the committee given the retrospective design.

### 4.2. Participants

All patients with an established diagnosis of Crohn’s disease or ulcerative colitis who underwent gastrointestinal surgery at the institution between April 2014 and November 2025 were eligible. The diagnosis of IBD was confirmed from the medical record by endoscopic biopsy, surgical resection, or histopathological diagnosis [22]. Patients without IBD, those undergoing anorectal surgery alone, and those with incomplete records were excluded.

A total of 158 operations were assessed for eligibility. Forty were excluded: 23 because the diagnosis of IBD could not be confirmed and 17 because records were incomplete or follow-up was shorter than 30 days. Of the 118 eligible operations (98 patients), 8 were performed through a dirty/infected wound (CDC wound class IV) with established intra-abdominal infection at the time of surgery. Because a subsequent infection in these operations cannot be distinguished from persistence of the infection present at operation, and because the antimicrobial administered may have represented treatment rather than prophylaxis, these 8 operations were excluded from the primary analysis and are reported separately; they are included only in a prespecified sensitivity analysis. The primary cohort therefore comprised 110 operations (Figure 1).

These 110 operations were performed in 91 patients (74 patients with one operation, 15 with two, and 2 with three). The unit of analysis was the operation; within-patient correlation was addressed in the analysis as described in Section 4.6.

### 4.3. Definitions and Outcomes

Surgical site infection was defined according to the surveillance criteria of the United States Centers for Disease Control and Prevention National Healthcare Safety Network and classified as superficial incisional, deep incisional, or organ/space, applying the NHSN definitions current at the time of data abstraction (2023 edition) retrospectively and uniformly to all operations regardless of the year of surgery [23]. Infection present at the time of surgery was recorded through the wound classification; all 8 operations classified as dirty/infected had established intra-abdominal infection at operation. The type of infection was determined from the diagnoses, signs, and symptoms recorded in the medical record, together with the treatment administered and the microorganisms isolated. The primary outcome was deep incisional or organ/space SSI. Any SSI was analyzed as a secondary outcome. Postoperative complications were graded using the Clavien–Dindo classification [24]; because SSI is itself a graded complication, Clavien–Dindo grade is reported descriptively within the infected group and was not entered as a candidate predictor.

All operations were followed for 30 days after surgery, and any SSI arising within that window was recorded. Postoperative follow-up was arranged case by case, with routine clinic review usually at about two weeks and again at about three months after operation, and earlier or additional visits as clinically indicated. Thirty-day SSI status was ascertained from the inpatient record, the two-week clinic review and any additional visits, and any emergency department attendance or readmission within 30 days. Operations for which 30-day status could not be established from these sources were excluded at the eligibility stage (17 of 158), so ascertainment was complete for the entire analyzed cohort.

### 4.4. Microbiological Methods and Classification of Expected Coverage

Specimens were processed in the clinical microbiology laboratory of King Abdulaziz University Hospital. Specimens were inoculated onto sheep blood agar, MacConkey agar, and chocolate agar and incubated at 35–37 °C in 5% CO_2_ for 18–48 h. Microorganisms were identified by matrix-assisted laser desorption/ionization time-of-flight mass spectrometry (MALDI-TOF MS). Antimicrobial susceptibility testing was performed on the VITEK 2 system (bioMérieux, Singapore), in use at the institution since 2012, supplemented by disk diffusion where required, and interpreted according to Clinical and Laboratory Standards Institute (CLSI) M100 breakpoints current in each year of the study. ESBL production was reported on the basis of the VITEK 2 ESBL test with CLSI phenotypic confirmation; methicillin resistance in *S. aureus* on the basis of cefoxitin screening; and carbapenemase production on the basis of the modified carbapenem inactivation method. Blood cultures were processed in an automated continuous-monitoring blood culture system. Resistance phenotypes were transcribed from the laboratory report for the index (first-reported) isolate of each infection; all reported co-isolates were recorded by species. Isolates were recorded as reported; no formal criteria were applied to distinguish causative microorganisms from colonizing or contaminating flora; wound swabs in particular are prone to this. Infections yielding only a coagulase-negative staphylococcus are identified as possible contaminants in Table 4.

Expected coverage of each culture-positive infection by the prophylactic regimen administered was classified per infection using every reported isolate. An isolate was classified as “expected not covered” if the laboratory report recorded an acquired resistance phenotype that ordinarily confers resistance to the agent administered (ESBL production to cefazolin and ceftriaxone, noting that current CLSI and EUCAST guidance reports cephalosporin susceptibility by measured MIC rather than by ESBL status, so this is an expectation rather than a determination; methicillin resistance to all beta-lactams other than anti-MRSA cephalosporins; carbapenemase production to carbapenems and piperacillin–tazobactam) or if the species is intrinsically resistant to the agent (*Enterococcus* to cephalosporins; *Enterococcus faecium* to carbapenems and piperacillin–tazobactam). An isolate was classified as “expected covered” if no acquired phenotype was recorded and the species is ordinarily susceptible to the agent. An isolate was classified as “indeterminate” if coverage could not be determined from these criteria, including ESBL-producing isolates against ciprofloxacin, *Acinetobacter* and *Enterobacter* species against the agent given, coagulase-negative staphylococci, and *Candida* species, which are not assessable against antibacterial prophylaxis. An infection was classified as “not covered” if any isolate was expected not covered, “covered” if all isolates were expected covered, and “indeterminate” otherwise. Because agent-specific susceptibility results were not uniformly retrievable, this is a phenotype-based expectation of coverage and not a susceptibility-based determination of discordance. The between-group comparison was repeated with indeterminate infections counted as covered, excluded, and counted as not covered.

### 4.5. Antimicrobial Prophylaxis

Antimicrobial prophylaxis was defined, following CDC guidance, as an agent administered preoperatively or intraoperatively to prevent SSI and discontinued within 24 h of operation; administration beyond that window is therapeutic rather than prophylactic [23].

Institutional practice was to administer a single preoperative dose within 60 min of incision, with intraoperative redosing for operations exceeding four hours. No written institutional prophylaxis protocol and no explicit criteria for regimen selection were in force during the study period; the regimen was selected by the operating surgeon, in practice according to the anticipated urgency and degree of contamination. In dirty/infected operations, the agent administered may have represented treatment of established infection rather than prophylaxis. The duration of postoperative antimicrobial administration was not retrievable from the available records.

### 4.6. Statistical Analysis

Analyses were performed using the Statistical Package for the Social Sciences, version 26 (IBM Corp., Armonk, NY, USA) and Python 3.12 with statsmodels 0.14. The distribution of continuous variables was assessed using the Shapiro–Wilk test. All departed significantly from normality with the exception of preoperative albumin (*p* = 0.066); for consistency of presentation, all continuous variables are reported as median with interquartile range and compared using the Mann–Whitney U test. Categorical variables are presented as counts and percentages and compared using the chi-square test, or the Fisher exact test where expected cell counts were below five.

The four covariates in the multivariable model (operative urgency, surgical approach, preoperative albumin, and IBD-directed therapy) were prespecified on the basis of the prior literature and clinical plausibility rather than selected by univariable screening, and their number was fixed in advance in relation to the expected number of events; univariable results are reported for description only. Because 17 patients contributed more than one operation, standard errors were clustered by patient (sandwich estimator), and a generalized estimating equation with an exchangeable working correlation was fitted as a check. Results are presented as odds ratios with 95% confidence intervals. Variables that are consequences rather than antecedents of infection, including length of hospital stay, Clavien–Dindo grade, and postoperative antimicrobial class, were excluded from the models. Model diagnostics comprised variance inflation factors, the Hosmer–Lemeshow test, and the area under the receiver operating characteristic curve. Given 22 events for four predictors, Firth penalized logistic regression was fitted as a bias-reduced alternative. A two-sided *p* value below 0.05 was considered significant.

No a priori sample size calculation was performed; all eligible operations during the study period were included. Proportions are reported with Wilson 95% confidence intervals. Missing data were handled by complete-case analysis. Diabetes status was missing for 2 operations and American Society of Anesthesiologists class for 2; all other model covariates were complete, so the multivariable models include all 110 operations. Prespecified sensitivity analyses comprised inclusion of the 8 dirty/infected operations, restriction to the first operation per patient, a three-predictor model omitting IBD-directed therapy, adjustment for study period (2014–2020 versus 2021–2025), and restriction to patients with Crohn’s disease.

The study was originally designed to describe SSI incidence and associated factors; the microbiological focus was developed during analysis. All risk-factor and microbiological analyses are therefore exploratory and hypothesis-generating.

## 5. Conclusions

This is the first description of the microbiology of SSI after surgery for IBD from the Arabian Peninsula. Deep and organ/space infection accounted for the majority of SSIs, and more than two-fifths of culture-positive infections involved a microorganism with a recorded acquired resistance phenotype. Among the culture-positive infections that followed correctly timed cephalosporin-based prophylaxis, nearly two-thirds yielded an isolate the agent was not expected to cover; this estimate rests on 11 culture-positive infections and describes discordance, not a causal effect. Low preoperative serum albumin was associated with deep infection in most model specifications, although the association weakened in the one-operation-per-patient analysis. Emergent surgery was not associated with deep infection once operations with established infection at the time of surgery were set aside. These findings support routine microbiological surveillance of SSI in IBD surgical programs and locally informed review of prophylaxis policy in settings where resistance among Enterobacterales is prevalent. They do not support empirical broadening of prophylaxis, and prospective, protocol-driven culture sampling with agent-specific susceptibility testing and standardized follow-up is required before any change in prophylaxis policy could be recommended.

## Figures and Tables

**Table 1:** Baseline characteristics of the primary cohort, by surgical site infection status (n = 110 operations in 91 patients).

Variable	Total, *n* (%)	SSI (*n* = 33)	No SSI (*n* = 77)	*p*
**Demographic**				
Age, years, median (IQR)	27 (22–41)	30 (25–47)	25 (22–38)	0.029 †
Male sex	66 (60.0)	22 (33.3)	44 (66.7)	0.400
Current smoker	7 (6.4)	4 (57.1)	3 (42.9)	0.194
Diabetes mellitus ‡	5 (4.5)	4 (80.0)	1 (20.0)	0.028
**Disease**				
Crohn disease	98 (89.1)	28 (28.6)	70 (71.4)	0.340
Ulcerative colitis	12 (10.9)	5 (41.7)	7 (58.3)	0.340
Perianal involvement	23 (20.9)	7 (30.4)	16 (69.6)	1.000
**IBD-directed therapy**				
Any therapy	77 (70.0)	18 (23.4)	59 (76.6)	0.025
Corticosteroids	35 (31.8)	9 (25.7)	26 (74.3)	0.656
Thiopurine or methotrexate	34 (30.9)	8 (23.5)	26 (76.5)	0.374
Biologic agent	55 (50.0)	15 (27.3)	40 (72.7)	0.678
Aminosalicylate	17 (15.5)	2 (11.8)	15 (88.2)	0.089
**Operative**				
Emergent operation	39 (35.5)	14 (35.9)	25 (64.1)	0.386
Open approach	63 (57.3)	24 (38.1)	39 (61.9)	0.037
Wound: clean/contaminated	70 (63.6)	20 (28.6)	50 (71.4)	0.672
Wound: contaminated	40 (36.4)	13 (32.5)	27 (67.5)	0.672
Stoma formation	28 (25.5)	7 (25.0)	21 (75.0)	0.635
Operative duration, min, median (IQR)	234 (176–313)	224 (176–305)	242 (181–320)	0.789 †
**Preoperative laboratory**				
Albumin, g/L, median (IQR)	35.0 (27.2–39.1)	29.2 (24.0–39.1)	35.1 (29.0–39.1)	0.057 †
White cell count, ×10^9^/L, median (IQR)	6.5 (5.0–8.7)	6.7 (5.0–10.2)	6.4 (5.0–7.8)	0.231 †
Hemoglobin, g/dL, median (IQR)	11.0 (9.7–12.6)	10.9 (9.7–12.3)	11.1 (9.7–12.8)	0.531 †

IQR, interquartile range; SSI, surgical site infection; IBD, inflammatory bowel disease. Percentages for demographic and operative rows are of the total column; percentages in the SSI and No SSI columns are row percentages within that category. † Mann–Whitney U test; all other comparisons by Fisher exact test. ‡ Missing for two operations. Four of five patients with diabetes developed infection (80.0%; 95% CI 37.6–96.4; Fisher exact *p* = 0.028); this estimate rests on five patients and should be interpreted accordingly. C-reactive protein was recorded for only 25 operations and is not tabulated.

**Table 2:** Microbiology of culture-positive surgical site infection in the primary cohort (*n* = 23).

Category	*n*	% of Culture-Positive
**Culture yield**		
Culture-positive	23	69.7 of all infections
Culture obtained, no growth	6	18.2 of all infections
No culture recorded	4	12.1 of all infections
**Specimen type**		
Wound swab	9	39.1
Deep tissue	8	34.8
Drain fluid	3	13.0
Intra-abdominal fluid	2	8.7
Blood	1	4.3
**Microorganisms isolated (28 isolates)**		
*Escherichia coli*	13	56.5
*Klebsiella* species	7	30.4
*Staphylococcus aureus*	2	8.7
Coagulase-negative staphylococci	2	8.7
*Candida* species	2	8.7
*Enterococcus faecalis*	1	4.3
*Streptococcus agalactiae*	1	4.3
Polymicrobial infections	5	21.7
**Recorded phenotype, index isolate**		
No acquired resistance phenotype recorded	13	56.5
ESBL-producing Enterobacterales (7 *E. coli*, 1 *K. oxytoca*)	8	34.8
Methicillin-resistant *S. aureus*	2	8.7
Any acquired resistance phenotype	10	43.5

ESBL, extended-spectrum beta-lactamase. Ten infections had no positive culture recorded and are included in the denominator for culture yield only. Phenotype refers to the index (first-reported) isolate; co-isolates are listed in Section 4.4.

**Table 3:** Preoperative antimicrobial prophylaxis in the primary cohort, by regimen (n = 110).

Regimen	n (%)	Emergent, n (%)	Contaminated Wound, n	SSI, %
Cefazolin + metronidazole	35 (31.8)	7 (20.0)	3	20.0
Ceftriaxone + metronidazole	30 (27.3)	6 (20.0)	9	23.3
Ciprofloxacin + metronidazole	28 (25.5)	13 (46.4)	14	39.3
Piperacillin–tazobactam or meropenem	17 (15.5)	13 (76.5)	14	47.1
Cephalosporin-based (combined)	65 (59.1)	13 (20.0)	12	21.5
Broader-spectrum (combined)	45 (40.9)	26 (57.8)	28	42.2

SSI, surgical site infection. All operations received the preoperative dose within 60 min of incision. Regimen selection was at the discretion of the operating surgeon; no institutional protocol or written selection criteria were in force. The higher infection rate in the broader-spectrum group reflects the concentration of emergent and contaminated operations in that group and should not be read as an effect of the antimicrobial agent. The eight dirty/infected operations, all of which received a non-cephalosporin regimen, are described in Section 2.8.

**Table 4:** Expected coverage of every culture-positive surgical site infection in the primary cohort by the prophylactic regimen administered (*n* = 23).

Regimen Group/Infection	Isolates Reported	Recorded Phenotype (Index Isolate)	Basis of Classification	Expected Coverage
**Cephalosporin-based (*n* = 11)**				
Ceftriaxone + metronidazole	*S. aureus*	MRSA	Acquired methicillin resistance	Not covered
Cefazolin + metronidazole	*S. aureus*	MRSA	Acquired methicillin resistance	Not covered
Cefazolin + metronidazole	*E. coli*	ESBL	Acquired ESBL	Not covered
Cefazolin + metronidazole	*E. coli*	ESBL	Acquired ESBL	Not covered
Cefazolin + metronidazole	*E. coli*, *E. faecalis*	ESBL	Acquired ESBL; *Enterococcus* intrinsically resistant to cephalosporins	Not covered
Ceftriaxone + metronidazole	*K. oxytoca*, *E. coli*	ESBL	Acquired ESBL	Not covered
Ceftriaxone + metronidazole	*E. coli*	ESBL	Acquired ESBL	Not covered
Ceftriaxone + metronidazole	*E. coli*	None recorded	No phenotype; Enterobacterales expected susceptible	Covered
Ceftriaxone + metronidazole	*E. coli*	None recorded	As above	Covered
Cefazolin + metronidazole	*K. pneumoniae*, *S. agalactiae*	None recorded	As above; *S. agalactiae* expected susceptible	Covered
Cefazolin + metronidazole	*K. pneumoniae*, *Candida* sp.	None recorded	As above; *Candida* not assessable against antibacterial prophylaxis	Covered ‡
**Broader-spectrum (*n* = 12)**				
Ciprofloxacin + metronidazole	*E. coli*	ESBL	Ciprofloxacin susceptibility of ESBL isolate not recorded	Indeterminate
Ciprofloxacin + metronidazole	*E. coli*	ESBL	As above	Indeterminate
Ciprofloxacin + metronidazole	Coagulase-negative staphylococcus	None recorded	Susceptibility not recorded; possible contaminant	Indeterminate
Piperacillin–tazobactam	Coagulase-negative staphylococcus	None recorded	As above	Indeterminate
Piperacillin–tazobactam	*Candida* sp.	None recorded	Not assessable against antibacterial prophylaxis	Indeterminate
Ciprofloxacin + metronidazole (×4)	*E. coli* (2) or *K. pneumoniae* (2)	None recorded	No phenotype; Enterobacterales expected susceptible	Covered
Piperacillin–tazobactam (×2)	*K. pneumoniae*; *E. coli* + *K. pneumoniae*	None recorded	As above	Covered
Meropenem	*E. coli*	ESBL	Carbapenem active against ESBL producers	Covered
**Summary**				
Cephalosporin-based				7 not covered/4 covered/0 indeterminate
Broader-spectrum				0 not covered/7 covered/5 indeterminate

ESBL, extended-spectrum beta-lactamase; MRSA, methicillin-resistant *Staphylococcus aureus*. Expected coverage was assigned per infection as “not covered” if any reported isolate was expected not to be covered on the basis of a recorded acquired-resistance phenotype or known intrinsic resistance, “covered” if all reported isolates were expected to be covered and no acquired phenotype was recorded, and “indeterminate” otherwise. This is a phenotype-based classification, not a susceptibility-based one: “no phenotype recorded” does not establish susceptibility to the agent given. ‡ Classified on the bacterial isolate; the *Candida* co-isolate was not assessed. Only index isolates carry a recorded phenotype; co-isolates were classified on species-level intrinsic resistance alone. The seven culture-positive dirty/infected infections are classified in Section 2.8.

**Table 5:** Multivariable logistic regression for surgical site infection in the primary cohort (110 operations in 91 patients; standard errors clustered by patient).

Variable	Any SSI aOR (95% CI)	*p*	Deep or Organ/Space SSI aOR (95% CI)	*p*
Emergent operation	1.27 (0.51–3.16)	0.614	2.39 (0.79–7.22)	0.122
Open approach	2.27 (0.92–5.63)	0.076	1.80 (0.62–5.23)	0.283
Preoperative albumin ≤ 35 g/L	2.35 (0.92–6.00)	0.074	3.59 (1.17–11.04)	0.025
No IBD-directed therapy	2.41 (1.00–5.80)	0.049	3.58 (1.29–9.97)	0.015

aOR, adjusted odds ratio; CI, confidence interval; SSI, surgical site infection; IBD, inflammatory bowel disease. Events: 33 for any SSI and 22 for deep or organ/space SSI. Wound classification (clean/contaminated versus contaminated) was not associated with infection and was not entered.

**Table 6:** Sensitivity analyses for deep or organ/space surgical site infection: adjusted odds ratios (95% CI).

Variable	Primary Model (n = 110, 22 Events) Cluster-Robust	Firth Penalized (n = 110, 22 Events)	First Operation per Patient (n = 91, 19 Events)	Including Dirty/Infected (n = 118, 30 Events) Cluster-Robust	Three-Predictor Model (n = 110, 22 Events)
Emergent operation	2.39 (0.79–7.22)	2.28 (0.84–6.19)	2.31 (0.75–7.13)	3.92 (1.36–11.28)	2.46 (0.87–6.94)
Open approach	1.80 (0.62–5.23)	1.70 (0.59–4.91)	2.26 (0.69–7.48)	2.11 (0.77–5.81)	2.19 (0.75–6.45)
Preoperative albumin ≤ 35 g/L	3.59 (1.17–11.04)	3.23 (1.07–9.72)	2.92 (0.80–10.59)	3.03 (1.10–8.33)	3.18 (1.14–8.91)
No IBD-directed therapy	3.58 (1.29–9.97)	3.35 (1.21–9.27)	3.57 (1.14–11.16)	3.59 (1.39–9.24)	—

IBD, inflammatory bowel disease. Cluster-robust standard errors by patient in the primary, dirty/infected-included, and three-predictor models. Firth penalized estimation applies a bias-reducing penalty for small samples. Confidence intervals excluding 1 in each column: primary model, albumin and no IBD-directed therapy; Firth, the same two; first operation per patient, no IBD-directed therapy; including dirty/infected, emergent operation, albumin, and no IBD-directed therapy; three-predictor model, albumin.

**Table 7:** The eight dirty/infected operations, reported separately from the primary analysis.

Regimen Administered	SSI Depth	Isolates Reported	Recorded Phenotype (Index Isolate)	Expected Coverage
Piperacillin–tazobactam	Organ/space	No growth	—	Not assessable
Ciprofloxacin + metronidazole	Organ/space	*E. coli*, *E. faecalis*	None recorded	Not covered (*Enterococcus* not reliably covered by ciprofloxacin)
Meropenem	Organ/space	*E. coli*, *E. faecium*, *A. baumannii*	None recorded	Not covered (*E. faecium* intrinsically resistant to carbapenems)
Piperacillin–tazobactam	Deep incisional	*K. pneumoniae*	None recorded	Covered
Ciprofloxacin + metronidazole	Deep incisional	*E. coli*	None recorded	Covered
Piperacillin–tazobactam	Organ/space	*E. cloacae*, *K. pneumoniae*	None recorded	Indeterminate (inducible AmpC)
Ciprofloxacin + metronidazole	Organ/space	*A. baumannii*, *C. freundii*	None recorded	Indeterminate (*A. baumannii* susceptibility not recorded)
Piperacillin–tazobactam	Organ/space	*E. coli*, *K. pneumoniae*	Carbapenemase	Not covered (acquired carbapenemase)

SSI, surgical site infection. All eight operations were emergent and involved established intra-abdominal infection at the time of surgery; the antimicrobial administered may have represented treatment rather than prophylaxis. Expected coverage was classified by the same criteria as Table 4.

## Data Availability

The datasets generated and analyzed during the current study are available from the corresponding author on reasonable request.

## Abbreviations

The following abbreviations are used in this manuscript:

SSI	Surgical site infection
IBD	Inflammatory bowel disease
ESBL	Extended-spectrum beta-lactamase
MRSA	Methicillin-resistant Staphylococcus aureus
CDC/NHSN	Centers for Disease Control and Prevention/National Healthcare Safety Network
aOR	Adjusted odds ratio
CI	Confidence interval
IQR	Interquartile range
GEE	Generalized estimating equation
